# Poly(levodopa)-Functionalized Polysaccharide Hydrogel Enriched in Fe_3_O_4_ Particles for Multiple-Purpose Biomedical Applications

**DOI:** 10.3390/ijms24098002

**Published:** 2023-04-28

**Authors:** Anna Michalicha, Anna Tomaszewska, Vladyslav Vivcharenko, Barbara Budzyńska, Magdalena Kulpa-Greszta, Dominika Fila, Robert Pązik, Anna Belcarz

**Affiliations:** 1 Chair and Department of Biochemistry and Biotechnology, Medical University of Lublin, Chodzki 1, 20-093 Lublin, Poland; anna.belcarz@umlub.pl; 2 Department of Biotechnology, Institute of Biology and Biotechnology, College of Natural Sciences, University of Rzeszow, Pigonia 1, 35-310 Rzeszow, Poland; 3 Independent Unit of Tissue Engineering and Regenerative Medicine, Chair of Biomedical Sciences, Medical University of Lublin, Chodzki 1, 20-093 Lublin, Poland; 4 Independent Laboratory of Behavioral Studies, Medical University of Lublin, Chodzki 4a, 20-093 Lublin, Poland; 5 Department of Inorganic Chemistry, Faculty of Chemistry, Institute of Chemical Sciences, Maria Curie-Skłodowska University, Maria Curie-Skłodowska Sq. 2, 20-031 Lublin, Poland

**Keywords:** poly(levodopa), Fe_3_O_4_ nanoparticles, biosafety, heat generation, zebrafish

## Abstract

In recent years, there has been a significant increase in interest in the use of curdlan, a naturally derived polymer, for medical applications. However, it is relatively inactive, and additives increasing its biomedical potential are required; for example, antibacterial compounds, magnetic particles, or hemostatic agents. The stability of such complex constructs may be increased by additional functional networks, for instance, polycatecholamines. The article presents the production and characterization of functional hydrogels based on curdlan enriched with Fe_3_O_4_ nanoparticles (NPs) or Fe_3_O_4_–based heterostructures and poly(L-DOPA) (PLD). Some of the prepared modified hydrogels were nontoxic, relatively hemocompatible, and showed high antibacterial potential and the ability to convert energy with heat generation. Therefore, the proposed hydrogels may have potential applications in temperature-controlled regenerative processes as well as in oncology therapies as a matrix of increased functionality for multiple medical purposes. The presence of PLD in the curdlan hydrogel network reduced the release of the NPs but slightly increased the hydrogel’s hemolytic properties. This should be taken into account during the selection of the final hydrogel application.

## 1. Introduction

Curdlan (β-1,3-glucan) is an unbranched polymer composed of glycosidic units. It shows specific gelling properties (capability to form high-set thermal irreversible hydrogels) and high water-sorption capacity, as well as satisfactory elasticity and mechanical resistance [1]. In the last decades, curdlan attracted notable attention in biomedical and pharmaceutical applications for its potential in drug delivery, anti-tumor, anti-HIV, and antimicrobial strategies, and even for its potential in the design of wound-dressing materials [2,3]. However, curdlan is capable of chemically binding therapeutically active agents only after its aggressive modifications via sulfation, carboxymethylation or phosphorylation [2] with simultaneous loss of its stable hydrogel structure and integrity. This reduces the utility of curdlan hydrogel for the preparation of functional biomaterials (e.g., wound dressings). Thus, mild modifications are often required to enhance the biomedical usefulness of curdlan [4].

Recently, the growing potential of highly adhesive polydopamine (PDA) coatings in biomaterials modification was highlighted [5]. PDA is produced by dopamine which polymerizes under slightly alkaline conditions in the presence of oxidants such as O_2_ or Cu^+2^, resulting in a highly adhesive coating [5]. So far, PDA layers have been successfully used to modify polysaccharide polymers [4], vascular devices made of surgical stainless steel [6], graphene nanosheets [7], and many other materials. PDA coatings were reported to positively affect the cellular response of coated surfaces [8] and the proliferation of endothelial cells in vitro [9]. In experiments performed by Liu et al., PDA coatings deposited on vascular stents promoted the growth of endothelial cells and showed antithrombotic and anti-inflammatory properties [10]. In addition, dopamine can easily oxidize to form a reactive quinone moiety that can bind various compounds possessing amino or thiol functional groups [11,12]. These properties were confirmed in our previous experiments concerning the curdlan hydrogels modified by PDA and PLD. We showed that both polycatecholamines can be formed within the curdlan hydrogel network before and after curdlan polymerization [4,13]. We also showed that a specific method of curdlan modification with PLD, based on L-DOPA introduction before curdlan thermal polymerization, results in relatively high nontoxicity for human fibroblasts and a lack of fibroblasts adhesion. PLD-modified curdlan was also capable of binding a significant quantity of gentamicin, resulting in significant antibacterial activity of the modified hydrogel [4].

What has expanded biomedical applications over time tremendously is the use of magnetic nanoparticles (MNPs), particularly as promising materials for tissue regeneration purposes [14]. The particles proved themselves sufficient and effective due to their great surface area, size-dependent superparamagnetic properties, precision tunability, as well as easy surface modification [15]. Since NPs can generate heat under the action of an alternating magnetic field (AMF), they are used for hyperthermia and heat-activated drug release [16]. Magnetic nanoparticles are divided into several classes—pure metals, metal oxides, and magnetic nanocomposite. For biomedical applications, the most popular are Co, Fe, Ni, Ti, iron oxide, and some ferrites. Among them, iron oxide nanoparticles are well known for biomedical purposes due to their low toxicity [17]. Another interesting feature of ferrites is related to the ability of light absorption and further energy dissipation into heat through net-phonon activation. What is particularly important is that the NIR (near infrared) spectral range can be utilized assuring localized and specific light interaction with ferrites since the biological systems show drastically minimized absorption within 750–1500 nm [18]. Both external and contactless stimulations can be used at the same time, and a synergic temperature effect can be induced that benefits from two distinctly different physical mechanisms of heat generation [19,20]. Moreover, ferrites can be additionally decorated with other metal nanoparticles, for example, silver ones, to enhance their biofunctionality.

Therefore, we found it interesting to verify whether PLD-modified curdlan can serve as a platform for the introduction of Fe_3_O_4_ particles to increase the biomedical potential of curdlan hydrogel. Fe_3_O_4_ particles were earlier found to bring some benefits to binary hydroxyapatite/curdlan hybrids designed for the therapy of bone tissue defects. Namely, the addition of magnetite nanoparticles increased the heating ability under the action of the AMF, 808 nm near-infrared laser radiation (NIR), and their synergic stimulation [21]. This strategy opens the new possibility of treating recurrent bone tissue cancers by biomaterials responding to heat-generating stimulants, especially since PDA was already reported to be used for the functionalization of Fe_3_O_4_ NPs as a platform for many aims [22,23]. Yang et al. showed the possibility of the production of a simple and intelligent sandwich nanocomposite. Ferrite nanoparticles were used as a carrier for two anti-cancer drugs, whereas PDA was used to avoid premature leakage of doxorubicin (DOX) before reaching the tumor site, increase photothermal therapy efficiency, and facilitate the integration of ZIF-8 (a type of organometallic backbone) [24]. The safety and potential effectiveness of the use of PDA-modified magnetic nanoparticles (NP Fe_3_O_4_@PDA) for the optimization of therapeutic strategies based on mesenchymal stem cells in the treatment of burn wounds were demonstrated [25]. Imran et al. obtained NP-based ferrofluids decorated via carbon core–shell. They reported an investigation of the electrical and thermal conductivity of both conctructs-Fe_3_O_4_ and Fe_3_O_4_@carbon (Fe_3_O_4_@C) core–shell nanoparticle (NP)-based ferrofluids via a chemical co-precipitation method followed by carbon coating as a shell over the Fe_3_O_4_ NPs via the hydrothermal technique [26]. The same research group presented an experimental method of preparing oil-based ferro-nanofluids with tunable thermal conductivity for heat transfer applications. Their experimental results proved that the method they used is more efficient for preparing ferro-nanoliquids with adjustable thermal conductivity as well as adjustable rheology suitable for heat transfer applications [27]. Yet, the available literature lacks characteristics of hydrogel constructs based on polydopamine and magnetite nanoparticles intended for biomedical applications such as wound treatment.

We hypothesized that PLD modification may increase the biomedical potential of curdlan hydrogel enriched with Fe_3_O_4_ nanoparticles and Fe_3_O_4_-Ag heterostructures, for example, to synthesize antibacterial wound dressings, biomaterials for thermal ablation of cancers, and other medical devices for regenerative medicine. We also found it interesting to verify the effect of the addition of PLD deposits on the stability of nanoparticles in the hydrogel matrix, on the hydrogels’ antibacterial activity, and on energy conversion for heat generation. Evaluation of these properties was also supported by the determination of cytotoxicity of biomaterial, safety in contact with human blood, mechanical properties, wettability, and other key features to confirm its suitability as material for biomedical applications.

## 2. Results and Discussion

### 2.1. Characterization of Nanoparticles and Heterostructures

**Figure 1:**
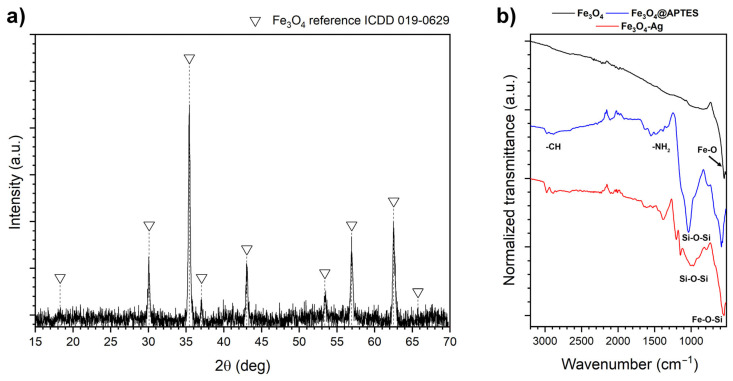
X-ray powder diffraction pattern of the Fe_3_O_4_ core nanoparticles (**a**) as well as FTIR-ATR spectra of the Fe_3_O_4_ nanocubes, Fe_3_O_4_@APTES core–shell, and Fe_3_O_4_-Ag heterostructures (**b**).

To characterize the nanoparticles synthesized for the functionalization of the polymer matrix in this study, X-ray powder diffraction technique (XRD), Fourier-transform infrared spectroscopy with attenuated total reflectance (FTIR), and Transmission Electron Microscopy (TEM) methods were used. XRD technique was used to evaluate the Fe_3_O_4_ structural properties. The measurement was carried out between 15 and 70° 2θ range. The obtained diffraction pattern (Figure 1a) after background correction and normalization was compared with the ferrite phase standard (card no. 019-0629). All recorded reflections match well with the reference ferrite structure (cubic *Fd*$\bar{3}m)$, and no additional peaks were observed, confirming the structural purity of the core particles.

FTIR-ATR spectra of the stock Fe_3_O_4_, Fe_3_O_4_@APTES core–shell nanoparticles, and Fe_3_O_4_-Ag heterostructures are shown in Figure 1b. As it can be noted, the Fe_3_O_4_ vibrational spectra consist of one intense peak located at around 550 cm^−1^ that is characteristic of the Fe–O vibration mode [28]. Deposition of the silica layer (APTES and TEOS source) on the surface of cubic Fe_3_O_4_ core particles leads to the appearance of several peaks at 1037 and 763 cm^−1^ attributed to the Si–O–Si and Si–OH structural unit vibrations, and 1557 and 1490 cm^−1^ were assigned to the amino functions while the band located at around 1630 cm^−1^ is due to the presence of OH groups [29,30,31]. Upon reduction of the silver cations, some shifts of the bands related to the vibrations of silica structural units were noted that is possibly attributed to the interaction of the Ag metallic particles with the silica shell. It was also observed that the vibration mode of the Fe–O bonds is shifted upon higher wavenumbers, being an indication of the appearance of Si–O–Fe modes [32].

**Figure 2:**
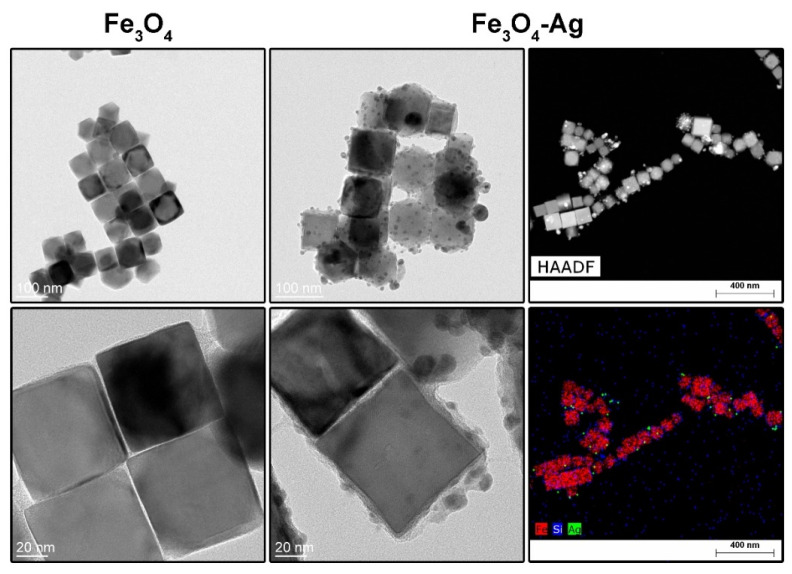
TEM images of the Fe_3_O_4_ nanocubes (**left**), Fe_3_O_4_–Ag heterostructures (**middle**), as well as STEM-HAADF imaging and STEM-EDS element mapping of the Fe_3_O_4_–Ag heterostructures (**right**).

TEM was used to estimate the size and morphology of the Fe_3_O_4_ nanoparticles and Fe_3_O_4_–Ag heterostructures while scanning transmission electron microscopy technique (STEM) with high-angle annular dark-field imaging (HAADF) was employed for element mapping in heterostructures. TEM imaging showed predominantly cubic morphology of the magnetic nanoparticles with an average size of 72.7 ± 7.9 nm (Figure 2 left). The average thickness of the silica coating was 7.9 ± 2 nm, whereas the size of Ag metallic particles was evaluated to be 8.2 ± 2.4 nm (Figure 2 middle) with a polyedric shape. From the element distribution maps, one can clearly see that the Ag particles are located within the outer part of the silica layer, while the silica formed a thin solid shell around magnetite core particles.

### 2.2. Characterization of Hydrogels (FTIR, NPs and Silver Ions Release, Heat Generation Effects, Mechanical Properties, and Wettability)

The as-synthesized nanoparticles were introduced to curdlan hydrogel, with and without the assistance of PLD. The effect of PLD on the physico-chemical properties of CR was reported elsewhere [4]. Hydrogels with both PLD and NPs were then characterized. The FTIR-ATR spectra were collected for all hydrogel samples (see Figure S1 in Supplementary Material). As one can note, an abundance of the very characteristic peaks related to the vibration of several structural curdlan elements can be found, namely at 3300 and 1645 cm^−1^ associated with water vibrations, at 2920, 1427, 1374, and 1301 cm^−1^ related to -CH-, -CH_2_-, -CH_3_- units, at 1257 cm^−1^ to C-OH, at 1205 cm^−1^ to C–O and C–O–C modes, at 1161 cm^−1^ ascribed to the C–O–C (ring) vibrations, at 1073, 1025, and 980 cm^−1^ to C–O modes, respectively [33]. Some unassigned vibrations below 600 cm^−1^ are difficult to ascribe to specific modes of pure curdlan material. It is important to note that all hydrogel samples showed the same FTIR spectral appearance. It was not possible to identify the vibrations related to the Fe_3_O_4_ (band around 550–580 cm^−1^) as well as PLD (range between 1200–1650 cm^−1^ and 3300 cm^−1^) since their content was low in comparison to the curdlan phase and, most probably, the modes are hidden and/or overlapped by highly intense vibrations of curdlan structural units.

**Figure 3:**
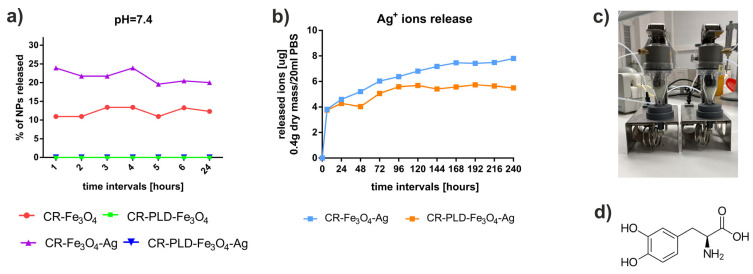
Nanoparticles release as % of total load (**a**) and Ag^+^ ions release (**b**) from curdlan hydrogels. SOTAX units of the release system used in experiments (**c**). Chemical structure of L-DOPA (**d**).

One of the most important features of NP-loaded biomaterials is the rate of release and the retention of particles entrapped within the 3D matrix. The release of Fe_3_O_4_ and Fe_3_O_4_–Ag particles was studied in a buffer of pH 7.4, typical for tissue liquids in a healthy organism, in the system presented in Figure 3a. It was found that during 5–6 h of the release, approx. 25% of the total number of Fe_3_O_4_–Ag particles were released from the curdlan hydrogels, whereas for Fe_3_O_4_, the release was lower (approx. 15%) (Figure 3a). Afterward, a drop in NP concentration in the release buffer was observed, which can be associated with NP aggregation or adsorption to the hydrogel fibers. Interestingly, no release of NPs was observed for all curdlan hydrogels modified with PLD (Figure 3a). Interactions between iron oxide particles and polydopamine were already reported in numerous articles [34,35,36]. Therefore, the observed phenomenon is likely to be related to the NP binding and stable entrapment in the PLD network during L-DOPA polymerization within the curdlan matrix.

Considering the antibacterial activity of designed matrices, the Ag^+^ release is essential. The cumulative profiles of Ag^+^ release from PLD-modified and unmodified hydrogels containing Fe_3_O_4_–Ag particles are presented in Figure 3b. Silver ions were gradually released to the medium during the first 5 days for both studied hydrogels, in a lower amount for the PLD-modified one. Then, the plateau was observed in the profile for CR–PLD–Fe_3_O_4_–Ag hydrogel, while further and slow release appeared for the CR–Fe_3_O_4_–Ag sample. The shorter time of the release of Ag^+^ ions from the PLD-modified sample is not conducive to the antibacterial properties of biomaterials. However, it must be taken into account that this phenomenon was studied in a semi-open system, and Ag^+^ ions released from CR–PLD–Fe_3_O_4_–Ag remained at the same level for the entire release period (10 days). The differences in the release profiles of ions can be explained. Positively charged Ag^+^ ions were likely to adsorb to the PLD deposits and then be transformed back into Ag nanoparticles. Silver ions were reported to undergo spontaneous reduction to metallic silver in contact with PDA [37]. For PLD, the tendency of Ag^+^ to be adsorbed on its surface is even higher due to the presence of a negatively charged free carboxyl group in the L-DOPA molecule in comparison with dopamine (Figure 3d). No release of iron ions was observed in collected samples. 

Another interesting feature of Fe_3_O_4_ NPs is stimuli-induced energy conversion with heat generation which can be useful in clinical applications for cancer tissue ablation. Contactless energy conversion measurements were performed as a function of the 808 nm laser output power on binary composites, i.e., CR–Fe_3_O_4_ and CR–Fe_3_O_4_–Ag and ternary materials after modification with L-DOPA: CR–PLD–Fe_3_O_4_ and CR–PLD–Fe_3_O_4_–Ag. The choice of such specific light wavelength was dictated by the fact that it covers the so-called I^st^ optical biological window (750–1000 nm) at which minimized absorption of the biological system can be found, leading to increased light penetration depth through tissues [18].

**Figure 4:**
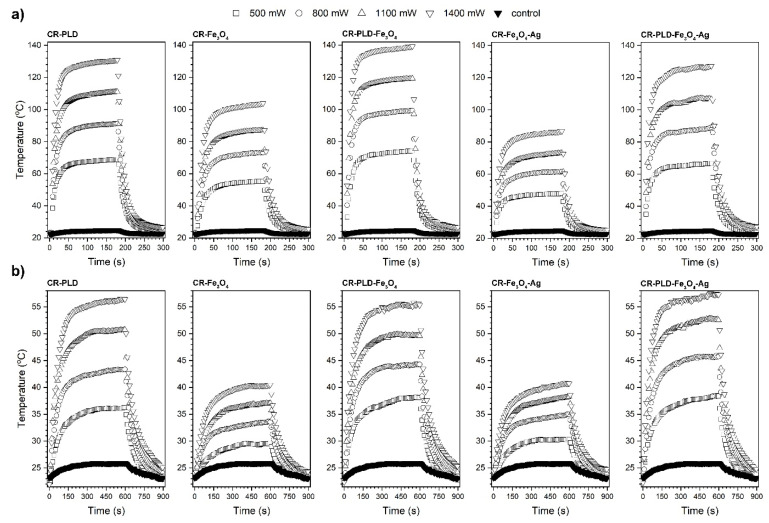
Heating curves of the dry (**a**) and soaked (**b**) nanocomposites as a function of the 808 nm laser stimulation. As a control sample, pure curdlan (CR) was used for comparison.

Heat generation effects were measured on dry binary or ternary composites as well as Ringer’s solution-soaked materials to mimic the tissue environment (Figure 4). The detailed characterization of the energy conversion behavior of the stock Fe_3_O_4_ nanocubes and Fe_3_O_4_–Ag under 808 nm photostimulation was presented by us elsewhere [38]. As one can observe, dry materials showed reasonable heat induction abilities. Even for the lowest laser power, 500 mW (1 W/cm^2^), the recorded temperature was above 43 °C during the first minute of measurement. For the maximum laser output power of 1400 mW (2.8 W/cm^2^), *T_max_* was around 104 °C for CR–Fe_3_O_4_ and 87 °C for CR–Fe_3_O_4_–Ag. The smaller *T_max_* of the latter sample is due to the fact that it contains less amount of the Fe_3_O_4_ while the Ag nanoparticles are not absorbing at this wavelength. In terms of achieved *T_max_*, the heating ability of the ternary composites, namely CR–PLD–Fe_3_O_4_ and CR–PLD–Fe_3_O_4_–Ag, was 139 °C and 127 °C, respectively. Such an increase in the maximum temperature for the ternary composite is directly related to the absorption properties of the L-DOPA, which shows relatively high absorption band intensity within this specific spectral region [39,40,41,42,43,44]. The soaking of composites with Ringer’s solution leads to a decrease in the recorded *T_max_* values in all cases. The maximum achievable temperature for the binary hybrids is around 40 °C with a strong dependence on laser power (or laser power density), while the addition of an additional absorber, i.e., L-DOPA, leads to improvement of the *T_max_* above 55 °C and gives a possibility of LOD reduction.

**Figure 5:**
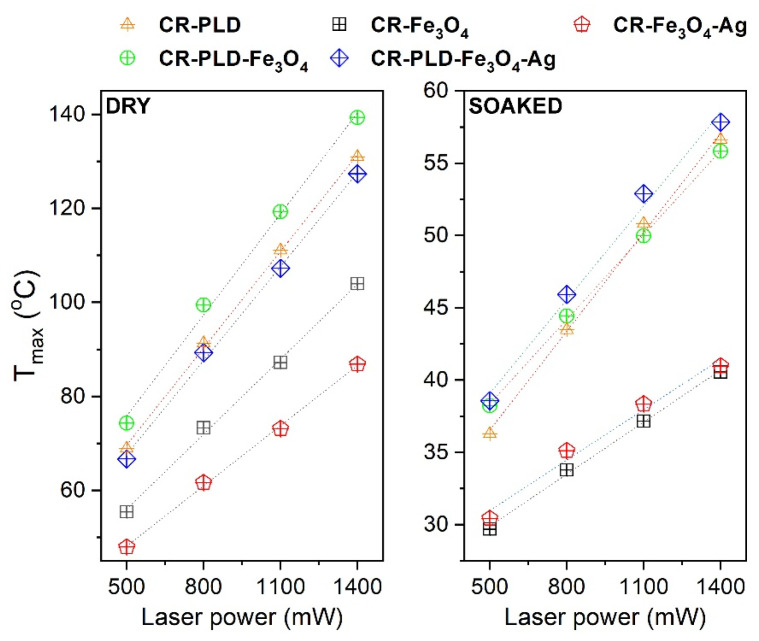
Laser power dependence of the dry and soaked binary and ternary composites.

The fact that the *T_max_* drops down is due to the presence of water with a high specific heat capacity (4.185 J/g °C). Analysis of the laser power dependence on the *T_max_* for dry and soaked hybrids is shown in Figure 5. As can be seen, the effect of this parameter is linear and may be used for the optimization of the *T_max_*. The addition of nanostructures and L-DOPA into ternary composites allows for obtaining therapeutic temperatures in soaked samples under contactless stimulation with NIR radiation and may have potential application in temperature-controlled regenerative processes as well as cancer therapies.

**Figure 6:**
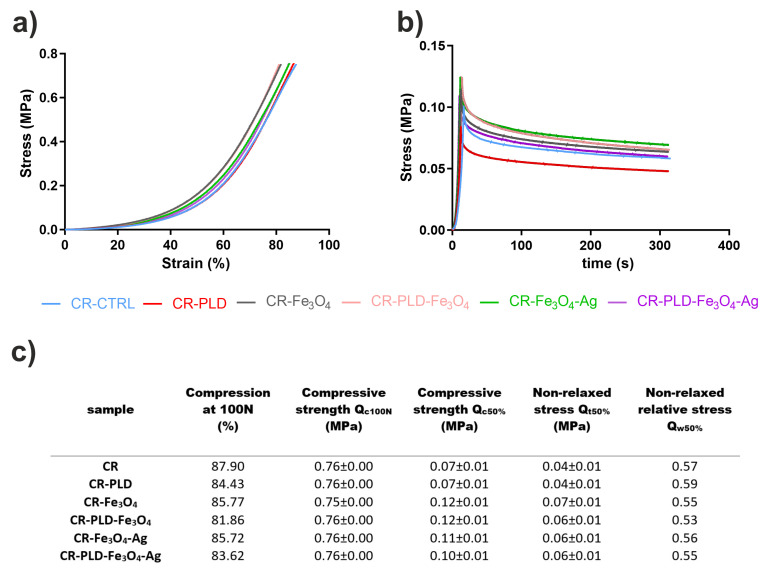
Stress–strain curves (**a**), relaxation curves (**b**), and mechanical parameters (**c**) of hydrogel samples (*σ_c_*_100N_—compressive strength measured as the maximum stress at 100 N, *σ_c_*_50%_—compressive strength as the maximum stress at 50 N, *σ_t_*_50%_—non-relaxed stress, and *σ_w_*_50%_—non-relaxed relative stress calculated as the ratio *σ_t_*_50%_*/σ_c_*_50%_).

The addition of iron oxide particles to polymers frequently reinforces the mechanical parameters of resulting composites [45]. For all curdlan hydrogel samples tested in this study, the shapes of stress–strain and relaxation curves were similar (Figure 6a,b). It could be concluded from the data obtained in mechanical tests that the addition of PLD slightly reduced the compression at 100 N load (from 87.9 MPa to 84.43 MPa for CR–CTRL, from 85.77 MPa to 81.86 MPa for CR–Fe_3_O_4_, and from 85.72 MPa to 83.62 MPa for CR–Fe_3_O_4_–Ag). However, the differences were very subtle, and this dependence was not reflected in other parameters (Figure 6c). Values of compressive strength measured as the maximum stress at 50% compression suggest that the addition of iron oxide particles can reinforce the hydrogels. Again, these results were not confirmed by other parameters. This may imply the lack of reinforcing effect of Fe_3_O_4_ particles on the hydrogel; however, this may result from the small dose of the particles in the samples.

Appropriate wettability of material affects desired biological response, ensuring adequate interaction between tissue and cells with the substrate [46]. It is one of the important wound dressing parameters since it affects bacteria adhesion and eukaryotic cell behavior on its surface [47]. One of the key features of external wound dressings is also their hydrophilic character, which enables them to absorb high amounts of exudates [48]. The material’s wetting characteristics were conducted using static contact angle measurements. The experiment showed that the contact angle formed on the top surface of each tested sample was lower than 90°, indicating their highly hydrophilic character. It should be noted that all material modifications positively influenced the samples’ hydrophilicity, reducing the contact angle formed by an ultrapure water droplet on the biomaterial surfaces compared to the control (Table 1). However, the presence of both PLD and NPs notably increased the hydrophilicity of pristine curdlan hydrogel: 48.78–59.27° in comparison with 77.22°. This may suggest that proposed modifications are beneficial for the biomedical properties of formed hydrogels.

### 2.3. Cytotoxicity and Toxicity in Fibroblasts and Zebrafish Model

**Figure 7:**
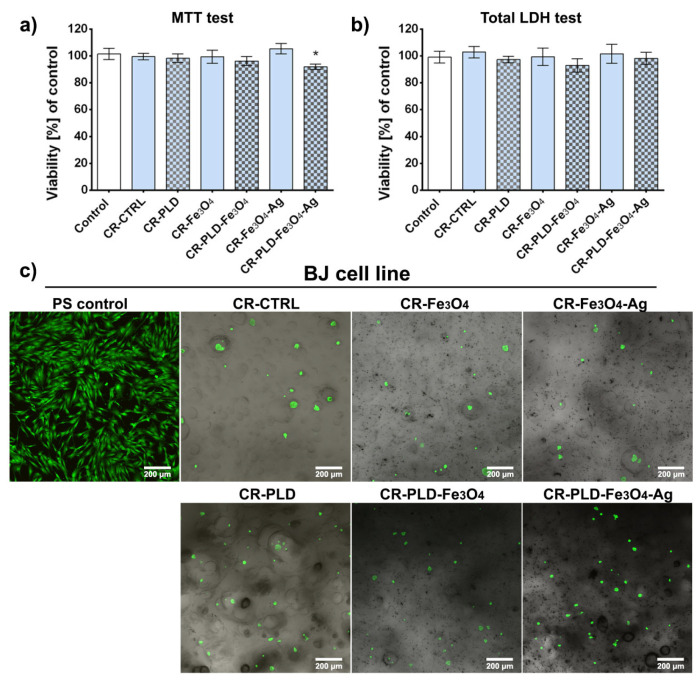
Cytotoxicity evaluation of the biomaterials: (**a**) MTT (cell metabolic activity) assay conducted according to ISO 10993-5 (* statistically significant results compared to the polypropylene control, *p*-value < 0.05, one-way ANOVA followed by Tukey’s test), (**b**) total LDH (cell number/biomass) assay conducted according to ISO 10993-5, (**c**) CLSM images of Live/Dead staining of BJ fibroblasts cultured on the produced materials for 48 h (green fluorescence represents viable cells, and red fluorescence represents dead cells).

The proposed application of modified poly(L-DOPA) and Fe_3_O_4_ NPs materials concerns the use of the above-mentioned materials as potential wound dressing for the treatment of skin wounds. The biocompatibility of biomaterials is one of the most crucial key features for wound dressing application since exposing the wound bed to a cytotoxic environment would significantly reduce the healing process or even lead to more serious consequences [49]. Therefore, MTT and total LDH tests were conducted to evaluate the cytotoxicity of the produced biomaterials against human skin fibroblasts. A lack of cytotoxicity of the materials was expected due to literature reports describing the biocompatibility of the used compounds [50,51,52]. The MTT cytotoxicity test by assessing fibroblast cell metabolism showed no cytotoxicity effect of tested extracts. Sample CR–PLD–Fe_3_O_4_–Ag caused a statistically significant reduction of cell viability compared to the polypropylene control; nevertheless, the viability was high and exceeded 91% (Figure 7a).

According to ISO 10993-5 standard, the decrease in cell viability below 70% indicates the cytotoxic effect of the tested sample. In the case of all tested extracts, cell viability was higher than 70%, indicating a lack of toxicity. Comparable results indicating high samples biocompatibility were obtained in the total LDH assay. It was revealed that all tested samples were non-toxic to the BJ fibroblast since the cell biomass for each material was high and exceeded 92% compared to the control (Figure 7b). No significant differences between the biomaterials and control were noted.

In the direct contact cytotoxicity test, BJ fibroblasts were cultured for 48 h and visualized using calcein-AM and propidium iodide (Live/dead staining). In the case of all tested materials, spherical, non-flattened, single, and non-attached skin fibroblasts were observed. Normal cell morphology and typical fibroblast growth were seen only on polystyrene (PS) surface (Figure 7c). No dead cells were noted, indicating that all produced materials were non-toxic but hindered fibroblast attachment and growth on the sample’s surface. This feature seems to be beneficial for biomaterials designed for wound dressings. Their lack of a supportive surface for cell adhesion proves that tested samples may be painlessly removed after potential use as an external wound dressing.

We also performed an assessment of the toxicity of our nanoparticle-modified biomaterials and the nanoparticles in ethanol solution used to modify these materials in the in vivo model. We opted for the zebrafish model as an alternative organism to the mammalian model.

**Figure 8:**
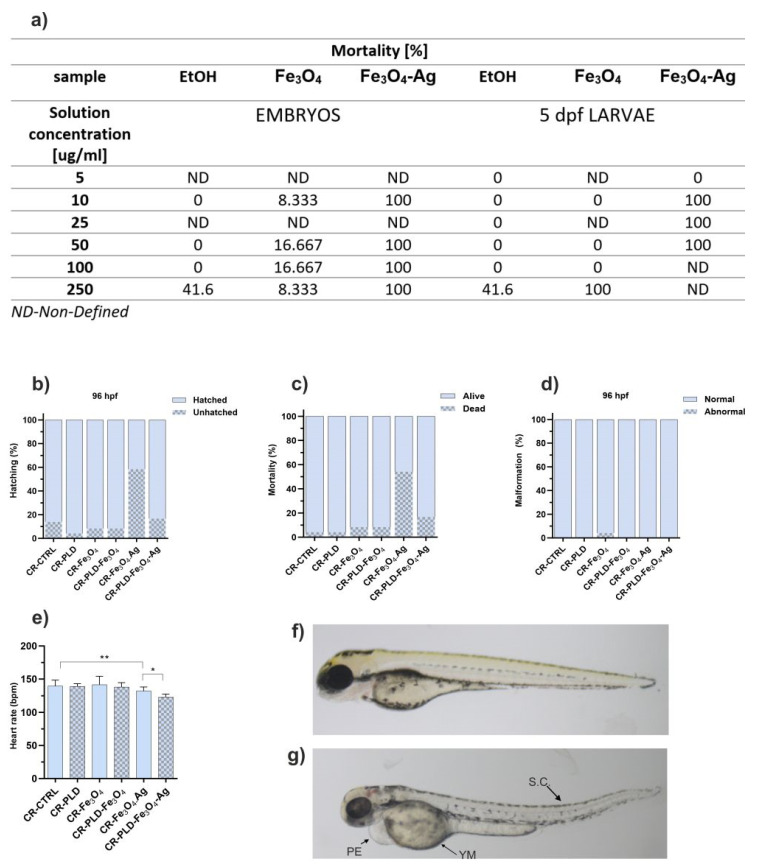
Mortality of *Danio rerio* embryo and 5 days post-fertilization (dpf) larvae in a concentration-dependent manner of nanoparticles exposure for 24 h (n = 24) (**a**) Effects of 96 h exposure to hydrogel extracts in zebrafish, (**b**) hatching rate, (**c**) a percentage of mortality, (**d**) percentage of morphological alterations, (**e**) heart rate estimated by beats per minute, (**f**) healthy *Danio rerio* larvae without malformations, (**g**) representative morphological alterations after incubation in CR–Fe_3_O_4_ solution in a 96 hpf larva indicated by arrows, PE—pericardial edema, SC—spinal curvature, YM—yolk sac malformation. Data are presented as means ± SD; n = 10; * *p* < 0.05, ** *p* < 0.01.

As shown in Figure 8a, free nanoparticles were found toxic for both zebrafish embryos and five5-dpf larvae (older and more environment-resistant organisms). Moreover, ethanol in a concentration equivalent to 250 µg/mL NPs also caused significant mortality (41.6%). This significantly limited the possible application of the nanostructures in solutions.

However, the observations of zebrafish embryos upon contact with extracts of NP-enriched hydrogels were much more promising. First, a high hatching ratio was observed for all extracts, with the exception of that of CR–Fe_3_O_4_–Ag (40% of hatched eggs; Figure 8b). Interestingly, we observed that the PLD modification of hydrogels increased the hatching rate in most cases. Second, the mortality of embryos was also low for all samples (2–8%), excluding the samples with Fe_3_O_4_–Ag. However, the presence of PLD reduced embryo mortality from 55% to 18% (Figure 8c). These markers are essential for biomaterials’ safety because they belong to the basic signs of toxicity in the zebrafish system. Importantly, all alive larvae did not show any sign of malformations (Figure 8d,f); only larvae incubated with extracts of CR–Fe_3_O_4_ showed some malformations (4.1%; Figure 8d,g). To investigate more specific toxic effects of tested extracts on the cardiovascular and central nervous systems, we analyzed changes in the heart rate of embryos. The extracts caused a decrease in the embryo’s heart rhythm (Figure 8e) in only two cases. Namely, a statistically significant decrease in the heart rate was observed between the CR–Fe_3_O_4_–Ag and CR–CTRL group (*p* < 0.01) as well as between CR–PLD–Fe_3_O_4_–Ag and CR–Fe_3_O_4_–Ag (*p* < 0.05). Overall, it is worth noting that hydrogels containing Fe_3_O_4_–Ag NPs, highly promising in most of the tested biomedical aspects, showed reduced toxicity against zebrafish embryos when modified with PLD.

### 2.4. Hemocompatibility

**Figure 9:**
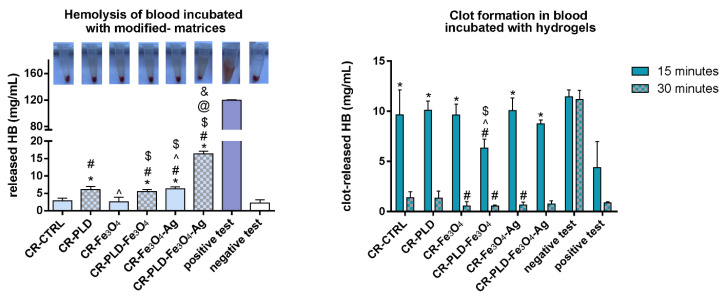
Hemolysis (**left**) and clot formation (**right**) in blood incubated with tested biomaterials. (*) symbol indicates statistically significant differences between the samples and positive test, (#) symbol indicates statistically significant results between CR–CTRL and the samples, (^) symbol indicates statistically significant results between CR–PLD and the samples, ($) symbol indicates statistically significant results between CR–Fe_3_O_4_ and the samples, (@) symbol indicates statistically significant results between CR–PLD–Fe_3_O_4_ and the samples, (&) symbol indicates statistically significant results between CR–Fe_3_O_4_–Ag and the samples; according to one-way ANOVA with post-hoc Dunnett’s test or post-hoc Tukey’s test (*p* < 0.05).

When designing hydrogels for medical applications, in particular, wound dressings, biomaterial–blood interactions must also be taken into account due to the frequent bleeding of the wounds. Therefore, human blood hemolysis and clot formation processes in contact with the tested materials were checked. Hydrogels modified only with Fe_3_O_4_ showed less blood hemolysis compared to the ones modified with Fe_3_O_4_–Ag, thus suggesting the hemolytic effect of silver deposits. Interestingly, slightly increased blood hemolysis was observed for the PLD-modified hydrogels, which is in agreement with our previous study results [13]. It should be noted that blood hemolysis, in case of blood contact with all samples except CR–PLD–Fe_3_O_4_–Ag, did not exceed 5% of the positive control, indicating the lack of hemolytic effect. Only CR–PLD–Fe_3_O_4_–Ag hydrogel showed hemolysis at the level of 13% (Figure 9), indicating that the type of potential application should be carefully selected for this hydrogel, excluding materials in constant contact with blood.

Concerning blood clot formation, for all hydrogels, we observed statistically significant inhibition of this process compared to the positive control after 15 min. However, after 30 min for all samples, the level of hemoglobin released from the clot was at a similar level as a positive control. Moreover, CR–Fe_3_O_4_, CR–PLD–Fe_3_O_4_, and CR–Fe_3_O_4_–Ag hydrogels showed increased pro-clotting properties than the control hydrogel (CR–CTRL) and reference sample (CR–PLD).

### 2.5. Antibacterial Properties

**Figure 10:**
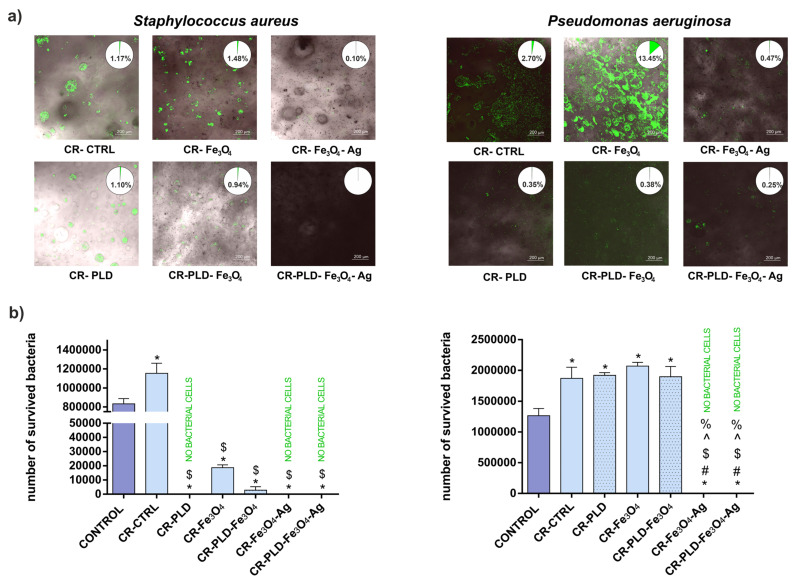
CLSM images of hydrogels incubated with bacteria for 2 h (**a**) and viability of bacteria in antimicrobial test (**b**) according to AATCC 100-2004 standard for porous materials, where: (*) symbol indicates statistically significant differences between the samples and the control for this test; (#) symbol indicates statistically significant results between CR–CTRL and the samples, ($) symbol indicates statistically significant results between CR–PLD and the samples, (^) symbol indicates statistically significant results between CR–Fe_3_O_4_ and the samples, (%) symbol indicates statistically significant results between CR–Fe_3_O_4_–Ag and the samples, according to one-way ANOVA with post-hoc Dunnett’s test or post-hoc Tukey’s test (*p* < 0.05). Insets in images presented in Figure (**a**) show the percent of image area covered by green fluorescence, indicating the presence of live bacteria.

Another feature that is essential for the biomedical usefulness of hydrogels is antibacterial activity, in particular bacterial adhesion prevention. Bacterial adhesion to biomaterials is the first step in biofilm formation. This devastating process leads to the appearance of highly resistant bacteria due to the presence of a protective barrier that screens the bacteria against the organism’s endogenous defense system or from external agents such as antibiotics [53,54]. As shown in Figure 10a, the presence of Fe_3_O_4_ particles did not prevent bacterial adhesion to the porous 3D structure of curdlan hydrogels. Actually, it seems even to increase the adhesion of *P. aeruginosa* cells. The observed enhancement of bacterial adhesion is in agreement with previous observations of strong bacterial attachment to the Fe_3_O_4_ NPs layer caused by the positive surface charge of the nanoparticles and the negatively charged surface of the bacteria [55]. All bacteria that adhered to the hydrogels were viable (as indicated by the green color). However, Fe_3_O_4_ particle decoration with Ag NPs drastically reduced the number of adhered bacteria in both tested strains (Figure 10a). This phenomenon was expected because silver ions are widely known antibacterial agents, and silver-based patented antimicrobial products and methods have increased in their number within the last decades [56]. The antibacterial effect of PLD was also noted, in particular for *P. aeruginosa*—the presence of polycatecholamine visibly reduced the number of hydrogel-adhered bacteria (Figure 10a). This effect was the most striking for CR–PLD–Fe_3_O_4_ samples versus CR–Fe_3_O_4_ ones. An observed phenomenon was expected because polydopamine, structurally related to PLD, was reported to reveal antibacterial activity [57]. In this experiment, the overall antibacterial activity of CR–PLD–Fe_3_O_4_–Ag hydrogel was higher than that of CR–Fe_3_O_4_–Ag, despite the observation that Ag^+^ release was lower for the former sample (Figure 3b). This suggests the beneficial effect of PLD content in tested hydrogels.

Bacterial-killing property observed in a test performed according to AATCC 100-2004 standard revealed that PLD deposition caused the nearly complete death of *S. aureus* (both for CR–PLD and CR–PLD–Fe_3_O_4_) but not *P. aeruginosa*. The presence of Fe_3_O_4_ particles in hydrogel reduced the viability of *S. aureus* without affecting *P. aeruginosa* strains. It is not surprising concerning previous reports. Diverse antibacterial activity of Fe_3_O_4_ NPs on Gram-negative and Gram-positive strains was noted in Gabryelian et al., depending on the concentration of particles [58]. However, a complete lack of bacterial viability appeared in hydrogels containing Fe_3_O_4_–Ag particles, both with and without PLD (Figure 10b). Considering the fact that hydrogel samples used in antimicrobial tests were washed for 1 week in water before experiments and only residual antibacterial activity was tested, the antibacterial activity of hydrogels enriched with PLD and Fe_3_O_4_–Ag particles is highly promising in terms of biomedical applications.

## 3. Materials and Methods

### 3.1. Materials

Curdlan powder (from *Alcaligenes faecalis*; cat. No. 281–80,531; DP 6790; average Mw 1100 kDa; specific rotation [A]^20^/_D_: +30 to +35; Cl^−^ content <0.5%, heavy metals content including Pb < 0.002%) was provided by Wako Chemicals (Osaka, Japan) and Tris (2-Amino-2-(hydroxymethyl)propane-1,3-diol) and L-DOPA (3,4-Dihydroxy-l-phenylalanine) by Sigma-Aldrich (St. Louis, MO, USA); all other reagents were of analytical grade from Avantor (Gliwice, Poland), unless stated otherwise.

### 3.2. Methods

The synthesis of the Fe_3_O_4_ nanoparticles and Fe_3_O_4_–Ag heterostructures.

#### 3.2.1. Fabrication of the Cubic MNPs

Fe_3_O_4_ nanoparticles were synthesized via the heat-up thermal decomposition technique. The reaction mixture was prepared in an acrylic glove box (GS Glove Box Systemtechnik GMBH P10R250T2) filled with inert gas (N_2_ 99.999%, Linde, Kraków, Poland) to protect sensitive reagents. The 2 mmol of iron acetylacetonate precursor (Fe(acac)3; 99.7%, Thermo Fischer Scientific, München, Germany) was mixed with 1.4 mL of oleic acid (4 mmol, OA, 90%, Sigma Aldrich, Poznań, Poland) and dissolved in 10 Ml of dibenzyl ether (BE, 98%, Sigma Aldrich, Poland) in a three-neck flask. The flask with a reaction mixture was further connected with a reaction set-up consisting of a reflux column, mechanical stirrer, gas line, and heating mantle equipped with a temperature controller (LTR 2500, Juchheim, Juchheim, Solingen, Germany) and a Pt-100 temperature sensor. Before the final heating, the mixture was degassed for 1 h at room temperature under a constant flow of N_2_. Subsequently, the temperature was raised to 285 °C and kept for 30 min. The resulting black powder containing MNPs was separated using a laboratory centrifuge and purified with 96% EtOH (Chempur, Piekary Śląskie, Poland). The Fe_3_O_4_ particle concentration was evaluated using a microbalance technique.

#### 3.2.2. Synthesis of the Fe_3_O_4_–Ag Heterostructures

Hybrid nanoparticles were synthesized in a two-step protocol using cubic magnetite as a core particle involving the previously established procedure [38]: (I) formation of the APTES shell on the surface of core MNPs; (II) deposition of plasmonic silver nanoparticles by a rapid silver cation reduction. Briefly, for the synthesis of APTES-covered Fe_3_O_4_, 50 mg of MNPs in ethanol dispersion was sonicated and transferred to the hexane using centrifugation and washing cycles (three times). After that, the 45 mL dispersion of cubic Fe_3_O_4_ was placed in an ultrasound bath for 2 h. The 2 mL of IGEPAL CO-520 (polyoxyethylene (5) nonylphenylether, Sigma Aldrich, Poznań, Poland) and 0.4 mL of ammonia solution (25% solution, 99%, Honeywell, Warsaw, Poland) were added after each other under constant sonication for 15 min. The flask with the mixture was attached to the set-up, and 100 μL of TEOS (Tetraethoxysilane, 99.9%, Thermo Scientific, Poland) was added under mechanical stirring for 1 h. Finally, 100 μL of APTES ((3-aminopropyl)triethoxysilane, 99%, Sigma Aldrich, Poland) and an additional 100 μL of TEOS were added and kept overnight for approximately 16 h under stirring. The product was purified by centrifugation-washing cycles with ethanol and acetone. The reduction of the silver cations on the magnetite core involved the use of 2 mg of the Fe_3_O_4_@APTES composite that was resuspended in 20 mL of deionized water using an ultrasound bath for 15 min. Afterward, four portions of 100 μL AgNO_3_ (99%, POCH, Gliwice, Poland) solution with a concentration of 1 mg/mL were added and constantly sonicated for 15 min. The glass flask was combined with a mechanical stirrer, and 760 μL of NaBH_4_ solution (1 mg/mL, 99%, Thermo Scientific, Warsaw, Poland) was added dropwise. The final product was purified three times with deionized water and once with ethanol. The Fe_3_O_4_–Ag heterostructures were redispersed in ethanol, and the concentration of hybrid nanoparticles was evaluated using the microbalance technique.

### 3.3. Characterization of Cubic Fe_3_O_4_ Nanoparticles and Fe_3_O_4_–Ag Heterostructures

Structural properties of the core Fe_3_O_4_ particles were characterized by means of XRD using a Bruker D8 Advance diffractometer equipped with the Cu lamp (*Κ_α_*_1_: 1.54 Å) and Ni filter for the removal of *Κ_α_*_2_ reflections. The recorded diffraction pattern was compared with reference card no. 019-0629 from the crystal structure database ICDD (International Centre of Diffraction Data-PDF database). The FTIR-ATR (Fourier-transform infrared spectroscopy with attenuated total reflectance) was utilized to study the presence of the silica shell and provide additional structural characterization of hybrid materials. Experiments were performed using a Thermo Scientific Nicolet iZ10 FTIR spectrometer equipped with a Smart Orbit Diamond ATR accessory. All spectra were recorded within the 4000–500 cm^−1^ range at room temperature.

The heterostructure size, morphology, as well as element mapping were carried out using a Tecnai Osiris X-FEG transmission microscope operating at 200 kV. A standard sample preparation technique was used for the TEM characterization. A droplet of nanoparticles suspended in ethanol (0.25 mg/mL) was deposited on a 200-copper mesh coated with a transparent carbon layer (EM Resolutions, Newcastle, UK). The resulting particle size of heterostructures, magnetic core, deposited silver, and silica shell thickness were evaluated with the help of ImageJ software (v. 1.8.0_1720).

### 3.4. Synthesis of Hydrogel Samples

**Figure 11:**
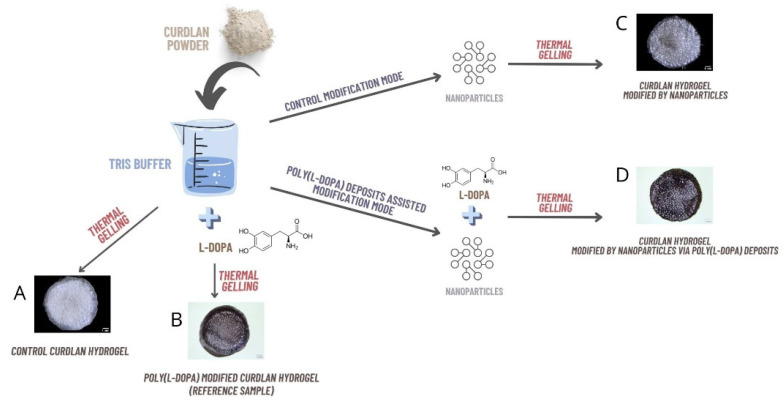
Samples synthesis process; (**A**) CONTROL CURDLAN HYDROGEL; (**B**) POLY(L-DOPA) MODIFIED CURDLAN HYDROGEL (REFERENCE SAMPLE); (**C**) CURDLAN HYDROGEL MODIFIED BY NANOPARTICLES; (**D**) CURDLAN HYDROGEL MODIFIED BY NANOPARTICLES VIA POLY(L-DOPA) DEPOSITS.

Hydrogels were synthesized according to the procedure described in Figure 1. Briefly, for control hydrogel (CR-CTRL; Figure 11A), the suspension of 0.4 g curdlan powder in 5 mL of Tris/HCl buffer pH 8.5 was stirred, transferred into glass tubes (ø 13 mm), polymerized at 93 °C for 15 min, and cooled. For reference PLD-modified samples (CR-PLD; Figure 11B), the suspension of 0.4 g curdlan in 5 mL 10 mM Tris/HCl buffer pH 8.5 was combined with L-DOPA monomer (2 mg/mL concentration), stirred for 10 min until L-DOPA was completely dissolved, transferred into glass tubes (ø 13 mm), polymerized at 93 °C for 15 min, and cooled.

NP-enriched samples (CR–Fe_3_O_4_ and CR–Fe_3_O_4_–Ag; Figure 11C) were prepared similarly as CR–CTRL samples, but an appropriate volume of NP solution was additionally added to the curdlan suspension (to obtain the final NP concentration 3 mg/g dry mass). NP-enriched and PLD-modified samples (CR–PLD–Fe_3_O_4_ and CR–PLD–Fe_3_O_4_–Ag; Figure 11D) were prepared similarly as CR–PLD ones, but an appropriate volume of NP solution was additionally added to the curdlan suspension (to obtain the final NP concentration of 3 mg/g dry mass), 15 min after L-DOPA introduction (to a final concentration of 2 mg/mL). For all NP-enriched hydrogels, the final suspensions were transferred into glass tubes (ø 13 mm), polymerized at 93 °C for 15 min, and cooled.

After cooling, all hydrogels were cut into 3 mm slices. Slices of CR–PLD, CR–PLD–Fe_3_O_4,_ and CR–PLD–Fe_3_O_4_–Ag hydrogels were incubated at 25 °C for 24 h in the air to enable PLD formation from L-DOPA. Then the slices of all hydrogels were washed 10 times in 100 mL DI H_2_O, frozen, and lyophilized (SRK, System Technik GMBK, Riedstadt, Germany).

Prior to cell cultures and zebrafish and antibacterial activity experiments, all hydrogels were sterilized by the ethylene oxide method in a paper/plastic peel pouch (sterilization for 1 h at 55 °C, aeration for 20 h). The FTIR-ATR spectra of all hydrogel samples were measured using the same apparatus as described earlier.

### 3.5. Nanoparticles and Ions Release

Nanoparticle release from hydrogel matrices (CR–Fe_3_O_4_, CR–PLD–Fe_3_O_4_, CR–Fe_3_O_4_–Ag, and CR–PLD–Fe_3_O_4_–Ag) was performed in a closed-loop system using a USP 4 compliant flow through the cell tester Sotax CE1 (Sotax, Basel, Switzerland). Lyophilized hydrogels were incubated with 20 mL PBS pH = 7.4 (proportion: 1 mL PBS/0.1 g hydrogel) at 37 °C and 1 mL/min laminar flow rate. In total, 0.3 mL of PBS was collected at a specified time point and replaced by fresh PBS. In collected PBS samples, the concentration of nanoparticles was measured spectrophotometrically at 370 nm and quantified based on calibration curves (for both NPs tested). The percentage of nanoparticles released from the samples over time was determined. The cumulative release profile of nanoparticles was calculated based on the results of 2 independent experiments (each in triplicate).

Ag^+^ ion release from hydrogels (CR–Fe_3_O_4_–Ag and CR–PLD–Fe_3_O_4_–Ag) was performed in a semi-open-loop system using sterile PBS pH 7.4 (proportion: 5 mL PBS/0.1 g dry weight of hydrogel sample) at 37 °C, with agitation (50 rpm, Innova 42, New Brunswick Scientific, Edison, NJ, USA). The extracts (15% of the total PBS volume) were collected daily and replaced by the same volume of fresh PBS. The experiment was performed for 2 independent experiments, each in triplicate. Then, Ag^+^ ion concentration was measured in daily collected extracts until detectable.

#### Ag^+^ Ion Concentration Measurements Methodology

The silver concentration in samples was determined using the Inductively Coupled Plasma Optical Emission Spectrometry, Varian 720-ES axial ICP-OES (Varian Inc., Palo Alto, CA, USA). The calibration curves were obtained using three standards (1; 2.5, and 5 mg/dm^3^) with the blank solution addition. The samples were not diluted before analysis. Moreover, the possible release of iron ions from hydrogel matrices (CR-Fe_3_O_4_-Ag and CR-PLD-Fe_3_O_4_-Ag) was investigated.

The ICP-OES operating conditions were as follows: power 1.0 kW, plasma gas flow rate 15.0 dm^3^/min, optical resolution 0.004 nm, pump speed 15 rpm, replicate read time 10 s, sample uptake delay 18 s, and replicates 3. Analytical lines for silver and iron were 328.068 nm and 259.940 nm, respectively. The limits of detection (LOD) for silver and iron were 0.005 mg/dm^3^.

### 3.6. Contactless Energy Conversion

The ability of the heat generation on composites under the action of NIR laser radiation was evaluated in a set-up equipped with a sample holder, continuous NIR laser device (808 nm), thermovision camera (FLIR T660, FLIR, Wilsonville, Oregon, USA), and insulated PS box to limit heat exchange. NIR radiation was delivered through 400 μm optical fiber, while laser power was verified and calibrated using an Ophir StarLite power meter with 10 A-PPS thermal sensors with beam track (Ophir, Jerusalem, Israel). The laser power used for sample stimulation was within 500–1400 mW which corresponded to the laser optical density (LOD) of 1–2.8 W/cm^2^. Heating curves were recorded by using a FLIR T660 camera. Data analysis was carried out with ResearchIR and OriginPro2019 software (9.6.0.172 (Academic), OriginLab Corporation, Northampton, MA, USA).

### 3.7. Mechanical Tests

Mechanical tests of all hydrogel samples were performed, as described elsewhere [59], using the EZ Test EX-SX universal testing machine (Shimadzu, Kyoto, Japan) equipped with the Trapezium program and a force sensor of 100 N. The compression test was carried out for hydrogel samples (ø = 13 mm; n = 10) completely soaked in PBS pH 7.4 with a crosshead rate of 5 mm/min. Measurements started after obtaining a force value of 0.05 N to eliminate gaps between the sample and the grips. The mechanical compression was carried out until a 100 N force was reached. The obtained results allowed us to determine compressive strength (*σc*_100N_), measured as the maximum stress at 100 N. The stress relaxation test was carried out by compression of the samples (ø = 13 mm; n = 10) until 50% of strain was reached. Then, the crosshead was stopped, and the force was measured for 5 min. The relaxation test allowed for the determination of compressive strength (*σc*_50%_), non-relaxed stress *σt*_50%_, and non-relaxed relative stress *σw_50%_* calculated as the ratio *σt*_50%_/*σc*_50%_.

### 3.8. Wettability Characterization

The wettability of the produced materials was evaluated using DSA 30 goniometer (Kruss GmbH, Hamburg, Germany). The static contact angle method consisting of the sessile drop technique of ultrapure water obtained from Milli-Q^®^ Water Purification System (Merck, Warsaw, Poland) was used in the test. At least three separate samples were tested for each type of material. All samples were evaluated in at least triplicate.

### 3.9. Cell Culture Experiments

Normal human skin fibroblasts (BJ cell line) were obtained from American Type Culture Collection ATCC-LGC Standards (Teddington, UK). BJ cells were cultured in a dedicated Eagle’s Minimum Essential Medium (EMEM, ATCC-LGC Standards, Teddington, UK) supplemented with 10% FBS (FBS, Pan-Biotech GmbH, Aidenbach, Bavaria, Germany), 100 μg/mL streptomycin, and 100 U/mL penicillin (Sigma-Aldrich Chemicals, Warsaw, Poland). The cells were maintained under standard conditions at 37 °C in a humidified atmosphere of 5% CO_2_ and 95% air.

#### Biocompatibility Evaluation

Cytotoxicity evaluation of produced biomaterials was carried out according to ISO 10993-5:2009 via the determination of BJ fibroblasts’ viability after exposure to 24 h biomaterials extracts. Due to the high absorption capacity and the swelling tendency of the tested materials [4,13], the ratio between sample weight and the volume of the extraction vehicle was reduced to 60 mg/mL, compared to the mentioned ISO standard. The BJ cells at a concentration of 2 × 10^4^ cells per well were seeded in flat bottom 96-multiwell plates and cultured for 24 h. Afterward, the medium was replaced with the as-prepared extracts obtained from biomaterials and polypropylene (negative control of cytotoxicity). After 24 h of incubation, cell metabolism evaluation was conducted using an MTT assay (Sigma-Aldrich Chemicals, Warsaw, Poland), and cell number was estimated using a total LDH test (Sigma-Aldrich Chemicals, Warsaw, Poland). The MTT test was performed according to the procedure described earlier [60]. The total LDH test was conducted according to the manufacturer’s instructions after the cell lysis. The results of both tests were expressed as the percentage of negative control of cytotoxicity. The cytotoxicity indirect tests using MTT and LDH assays were repeated in three independent experiments.

A direct contact cytotoxicity test using a Live/Dead Double Staining Kit (Sigma-Aldrich Chemicals, St. Louis, MO, USA) was also conducted. All biomaterials in the form of discs approx. 3 mm thick and 7 mm in diameter were sterilized by ethylene oxide, placed in the wells of a 48-multiwell plate, and presoaked in a complete culture medium at 37 °C. Then, human skin fibroblasts were seeded directly on the tested samples in 500 µL of the medium at a concentration of 2 × 10^5^ cells/mL. A PS plate well served as a control. After 48 h, BJ fibroblasts were stained with calcein-AM and propidium iodide following manufacturer protocol and analyzed using a confocal laser scanning microscope (CLSM, Olympus Fluoview equipped with FV1000, Olympus Corporation, Tokyo, Japan).

### 3.10. In Vivo Experiments—Danio rerio Model

*Danio rerio* of the AB strain was maintained at the Experimental Medicine Center, the Medical University of Lublin, Poland, at 28.5 °C, on a 14/10 h light/dark cycle under standard aquaculture conditions. Fertilized eggs were collected via natural spawning. Embryos were reared in embryo medium E3 (pH 7.1–7.3; 17.4 µM NaCl, 0.21 µM KCl, 0.12 µM MgSO_4_, and 0.18 µM Ca(NO_3_)_2_) in an incubator at 28.5 °C. Immediately after the experiment, larvae were killed by immersion in 15 μM tricaine solution. All details were described by Michalicha et al. 2021 [4]. All experiments were carried out in accordance with the National Institute of Health Guidelines for the Care and Use of Laboratory Animals and the European Community Council Directive for the Care and Use of Laboratory Animals of 22 September 2010 (2010/63/EU). Experiments with larvae older than 120 hpf were approved by the Local Ethics Committee in Lublin, Poland (Permission No: 44/2022). For the experiment with larvae up to 120 hpf, the agreement of the Local Ethical Commission was not required.

The fish embryo toxicity (FET) test was based on the modified OECD Guidelines for the Testing of Chemicals (OECD, 2013). Embryos, not later than 90 min post-fertilization, were investigated under a light microscope (Stemi 508, Zeiss, Oberkochen, Germany), and 24 viable fertilized embryos were selected and transferred to 96-well plates within 3 hpf. Embryos were individually incubated in 200 µL of tested compounds or control solutions. The embryos were exposed to NP suspensions or extracts of tested hydrogels (0.1 g of dry weight/1 mL E3 medium, 24 h, 37 °C) for 96 h. Embryos were observed every 24 h under a stereomicroscope, and the survival, hatching rate, and developmental abnormalities were recorded. At 96 h post-fertilization (hpf), the number of dead embryos, malformations, and hatching rate was scored, and the heart rate was measured. The larvae were equilibrated at room temperature for 30 min, and the heartbeats were counted under a stereomicroscope for 15 s. Obtained values were multiplied by four to obtain the number of beats per minute (bpm).

### 3.11. Blood Compatibility Tests

For blood compatibility tests, human-citrated blood was obtained from a healthy volunteer (procedure approved by the Bioethics Committee at the Medical University of Lublin, no KE-0254/258/2020). Its total hemoglobin and plasma hemoglobin concentration (measured by the reaction with Drabkin reagent and appropriate calibration curve, using 96-well plates and Synergy H4 hybrid microplate reader, Biotek, Winooski, Vermont, USA) were 1.38 mg/mL and 0.13 mg/mL, respectively. As a negative control, 30 mg ± 2 mg of HDPE (high-density polyethylene, Sigma-Aldrich, St. Louis, MO, USA) pieces was used in the hemolysis test and non-activated Ca^2+^-free whole blood in the blood clot formation test. The positive control in the hemolysis test contained 0.1% Triton X-100 test, while in the blood clot formation test: 30 mg ± 2 mg of HDPE pieces.

Hemolysis and blood clot formation tests were performed on 30 mg ± 2 mg samples of all hydrogels (each variant in triplicate), as described elsewhere [4].

### 3.12. Antibacterial Activity Evaluation

#### 3.12.1. Bacterial Strains and Maintenance

Two reference bacterial strains (*Staphylococcus aureus ATCC 25923*; *Pseudomonas aeruginosa ATCC 27853*) were grown at 37 °C for 20–24 h in Mueller–Hinton Agar medium (Biomaxima, Poland). Then the bacteria were scratched and suspended in a sterile physiological solution or Mueller–Hinton (M-H) broth (Biomaxima, Lublin, Poland) to appropriate density.

#### 3.12.2. Antibacterial Activity Test (Based on the Standard: AATCC Test Method 100-2004)

Antibacterial activity evaluation was performed as described earlier [4]. Briefly, all hydrogels were first washed in distilled water to remove the loosely bound drug (7 days, 100 mL exchanged daily), then placed on sterile Petri dishes (in quadruplicate) and treated with bacterial suspension (1.5 × 10^5^ CFU/mL) of each strain, prepared in 250-fold diluted M-H broth. Before the experiment, the volumes of bacterial suspension were calculated in accordance with the absorption capacity for each hydrogel. All of the samples were then incubated at 37 °C for 24 h, then transferred to sterile 0.9% NaCl (in volume 100-fold larger than the volume of bacterial suspension absorbed by samples) and vigorously shaken (1 min) to elute the bacterial cells from hydrogel network. The same volume of bacterial inoculation incubated without contact with hydrogels served as a control. Samples of the collected eluate were plated (in triplicate) onto M-H agar using an automatic plater (EasySpiral Dilute, Interscience, Saint-Nom-la-Bretéche, France), and the plates were incubated at 37 °C for 48 h. CFUs were then counted for each plate (Scan 300 counter).

#### 3.12.3. Bacterial Adhesion Test

The test was performed according to the protocol described earlier [4,13]. Briefly, the samples of hydrogel were incubated in 1 mL of bacterial suspensions (approx. 3.0 × 10^8^ cells/mL; in M-H broth) of each strain (2 h, at 37 °C,100 rpm, Innova 42, New Brunswick Scientific, Edison, NJ, USA). Afterward, non-adhered bacteria were gently washed away with 0.9% NaCl (10 min, 50 mL, 4 times). Washed samples were incubated with a Viability/Cytotoxicity Assay Kit for Bacteria Live & Dead Cells (Biotium, Fremont, CA, USA) in 0.9% NaCl (at R/T, 15 min, in darkness), according to the manufacturer’s instruction. After staining, the samples were washed in 0.9% NaCl to remove the non-absorbed dye. Adhered bacteria were visualized by LSCM (Olympus Fluoview FV1000; Olympus, Japan equipped with FV10 ASW 4.2 program).

## 4. Conclusions

Our observations showed that among the tested samples, hydrogel modified both with PLD and Fe_3_O_4_–Ag (CR–PLD–Fe_3_O_4_–Ag) showed the most promising features. They include stable entrapment of nanoparticles within the curdlan network, beneficial antibacterial effect, low zebrafish larvae mortality, a lack of cytotoxicity, significant wettability, and the most significant stimuli-induced energy conversion with heat generation, which are desired features in the designing process of biomaterials, despite some hemolytic activity. This suggests that this hydrogel construct can be considered promising for biomedical applications, excluding those associated with prolonged contact with blood. However, some of these effects were also observed for reference hydrogel (CR–PLD). The question is whether it is profitable to create a more complex system containing magnetic heterostructures when relatively similar parameters are obtained by a hydrogel modified only with PLD. However, it should be taken into account that polycatecholamines belong to biodegradable polymers. Therefore, there is no guarantee that the PLD provides a long-term therapeutical effect. In this light, the addition of nanoparticles with an enhanced antibacterial effect and significant stimuli-induced energy conversion to the biodegradable polymer matrix is a rational solution. Importantly, fabricated biomaterials are characterized by a lack of supportive surface for cell adhesion, which proves that tested samples may provide painless removal after potential use as an external wound dressing.

Due to several beneficial properties, curdlan hydrogels modified with functional nanoparticles via PLD deposits seem to be a promising proposal in the production of biomaterials for a wide range of medical applications. Therefore, the presented concept deserves further research of a wider scope.

## Figures and Tables

**Table 1:** The contact angle measurements of the tested materials.

Biomaterial Designation	Contact Angle [°]
CR–CTRL CR–PLD CR–Fe_3_O_4_ CR–PLD–Fe_3_O_4_ CR–Fe_3_O_4_–Ag CR–PLD–Fe_3_O_4_–Ag	77.22 ± 8.24 58.38 ± 9.07 57.04 ± 5.49 51.90 ± 5.23 59.27 ± 11.43 48.78 ± 9.57

## Data Availability

Not applicable.

## Supplementary Materials

The following supporting information can be downloaded at: https://www.mdpi.com/article/10.3390/ijms24098002/s1.
