# Supplementary material for: Poly(levodopa)-Functionalized Polysaccharide Hydrogel Enriched in Fe3O4 Particles for Multiple-Purpose Biomedical Applications
Source: Int J Mol Sci. 2023 Apr 28;24(9):8002. doi: 10.3390/ijms24098002 (PMC10178464; doi:10.3390/ijms24098002)
Supplement: Supplementary file 1 [file ijms-24-08002-s001.zip › ijms-2304101-supplementary.pdf]

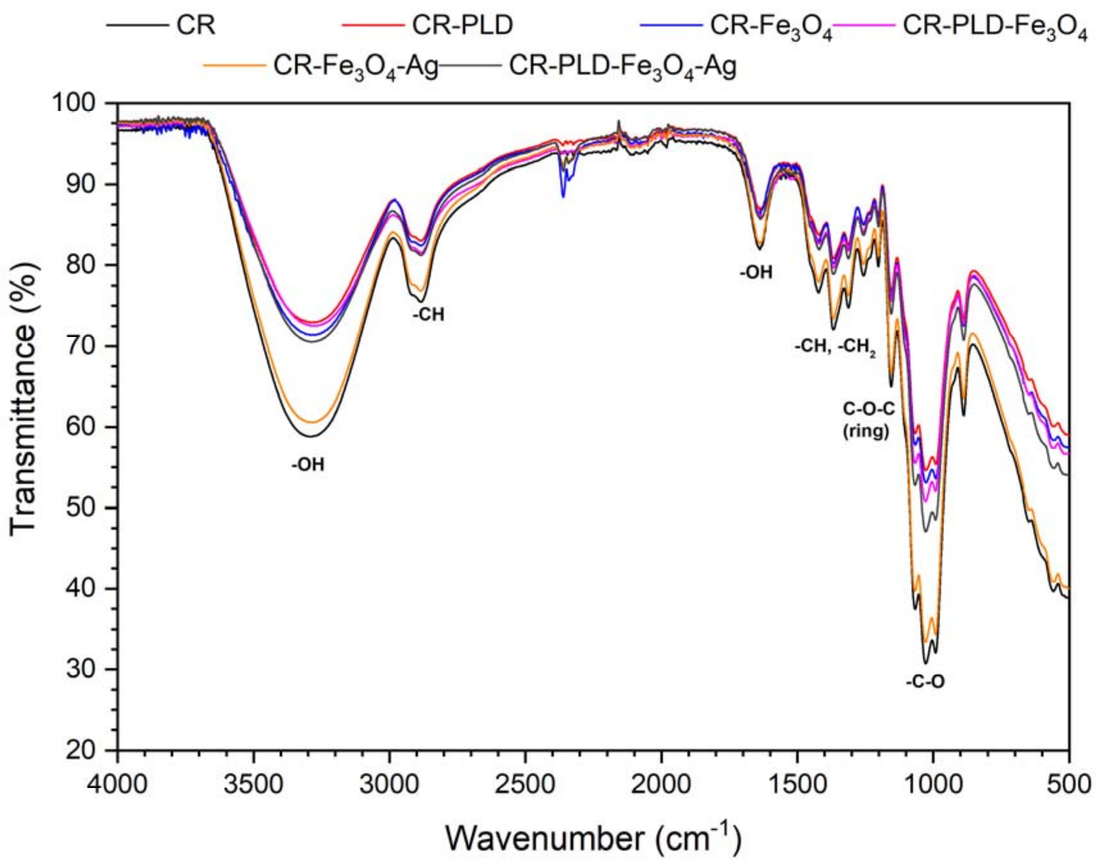

Figure S1. FTIR- ATR spectra of curdlan based hydrogels including CR-CTRL; CR-PLD; $CR-Fe_3O_4$; $CR-PLD-Fe_3O_4$; $CR-Fe_3O_4-Ag$; $CR-PLD-Fe_3O_4-Ag$ samples.
